# AI-guided refinement of coronary revascularization need in patients suspected of acute coronary syndrome

**DOI:** 10.1093/ehjdh/ztaf106

**Published:** 2025-10-06

**Authors:** Manuel Sigle, Diana Heurich, Wenke Faller, Meinrad Gawaz, Karin Anne Lydia Mueller, Andreas Goldschmied

**Affiliations:** Department of Cardiology and Angiology, University Hospital Tübingen, Eberhard Karls University Tübingen, Otfried-Müller-Str. 10, Tübingen 72076, Germany; Department of Cardiology and Angiology, University Hospital Tübingen, Eberhard Karls University Tübingen, Otfried-Müller-Str. 10, Tübingen 72076, Germany; Department of Cardiology and Angiology, University Hospital Tübingen, Eberhard Karls University Tübingen, Otfried-Müller-Str. 10, Tübingen 72076, Germany; Department of Cardiology and Angiology, University Hospital Tübingen, Eberhard Karls University Tübingen, Otfried-Müller-Str. 10, Tübingen 72076, Germany; Department of Cardiology and Angiology, University Hospital Tübingen, Eberhard Karls University Tübingen, Otfried-Müller-Str. 10, Tübingen 72076, Germany; Department of Cardiology and Angiology, University Hospital Tübingen, Eberhard Karls University Tübingen, Otfried-Müller-Str. 10, Tübingen 72076, Germany

**Keywords:** Machine learning, ACS, Coronary revascularization, Prehospital

## Abstract

**Aims:**

Overdiagnosis in patients suspected of acute coronary syndrome (ACS) leads to unnecessary coronary angiographies, particularly in cases with non-specifically elevated troponin (Trop) levels. We established machine learning (ML) models integrating sequentially available prehospital and in-hospital variables to improve early prediction of the need for coronary re while minimizing overdiagnosis.

**Methods and results:**

Retrospective cohort study analysing patients with suspected ACS from 2016 to 2020. Machine learning models were trained using data available at different diagnostic time points, including prehospital assessment, arterial blood gas analysis, full laboratory results, and sequential Trop measurements. A total of 2756 patients were included, identified through emergency physician protocols for ACS-related complaints. Patients with incomplete data or prehospital mortality were excluded.

Model performance improved with additional diagnostic data. Model 1 (prehospital data only) achieved an area under the receiver operating characteristic (AUROC) of 0.76 (95% confidence interval [CI] 0.72–0.79), while Model 4 (including sequential Trop testing) reached 0.87 (95% CI 0.83–0.91). Adding early hospital diagnostics (Model 2) significantly improved accuracy compared with Model 1 (0.65 vs. 0.78). Sequential Trop testing in Model 4 did not substantially enhance performance compared with single Trop testing in Model 3 (AUROC 0.87, 95% CI 0.83–0.91 vs. 0.86, 95% CI 0.82–0.91). Misclassification analysis revealed that underdiagnosed patients were typically older females with dyspnoea and known coronary artery disease but no ST-elevations. Overdiagnosed patients had higher body mass index, ST-elevations, regional wall motion abnormalities, and impaired left ventricular ejection fraction but lacked significant sequential Trop elevation.

**Conclusion:**

Prehospital assessments combined with early in-hospital diagnostics provide reliable stratification of coronary intervention need, potentially optimizing clinical decision-making and resource utilization.

## Graphical Abstract

**Graphical Abstract:**
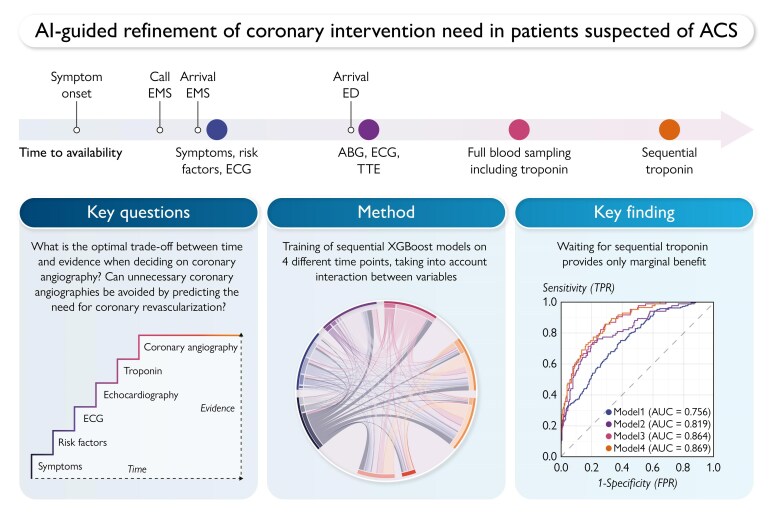

## Introduction

Acute coronary syndrome (ACS) remains a leading cause of morbidity and mortality worldwide, demanding prompt and accurate diagnosis to ensure timely treatment. Current clinical decision-making is based on prehospital assessments, such as symptoms, risk factors, and electrocardiography (ECG), as well as in-hospital diagnostic tools, including echocardiography, blood sampling, and finally invasive coronary angiography. However, this approach frequently results in overdiagnosis, particularly in patients with non-specifically elevated troponin (Tro) levels, leading to unnecessary coronary angiographies. These invasive procedures not only expose patients to potential complications but also strain healthcare resources.

Troponin, a cornerstone biomarker for ACS diagnosis, is highly sensitive but lacks specificity.^1,2^ Non-cardiac conditions such as renal dysfunction, sepsis, or pulmonary embolism can also cause Trop elevation, complicating the differentiation of true ACS from other conditions. Consequently, many patients undergo coronary angiography despite lacking an actual need for revascularization, underscoring a critical gap in the diagnostic process.

The integration of machine learning (ML) into clinical workflows offers a promising solution by identification of patterns and interactions among clinical variables of complex datasets that are not readily apparent through traditional analyses. Recent studies have highlighted the potential of ML to enhance diagnostic accuracy,^3–6^ but none have explored its ability to dynamically integrate sequentially available data from prehospital and in-hospital settings to guide decisions on coronary revascularization.

This study aimed to develop a series of ML models trained on data available at different diagnostic time points, ranging from prehospital assessments to advanced in-hospital diagnostics. By progressively incorporating variables such as arterial blood gas (ABG) parameters, echocardiography, comprehensive laboratory results, and sequential Trop measurements, we sought to refine predictions of PCI need while minimizing overdiagnosis. Additionally, we analysed patient characteristics associated with misclassification to identify predictors of over- and underdiagnosis.

## Methods

### Study population

This retrospective, single-centre cohort study analysed prehospital and in-hospital records from patients with a prehospital suspect of ACS between August 2016 and October 2020. Arterial blood gas was a prehospital working diagnosis made by emergency medical service personnel based on clinical presentation. Specifically, ACS was defined as new-onset or worsening chest pain with or without concomitant ECG changes. To identify such patients, care protocols from emergency physicians (EPs) were filtered for the diagnoses ‘ST-elevation myocardial infarction (STEMI)’, ‘non-ST-elevation myocardial infarction (NSTEMI)’ and ‘chest pain’. Exclusion criteria included an age below 18, prehospital death, ambulatory treatment, patient-declined transportation, and transportation to a medical facility other than the Emergency Department of the University Hospital, Tübingen, Germany.

Relevant prehospital patient data were transferred manually from the prehospital treatment protocols. In-hospital data were taken retrospectively from our clinical database. Myocardial infarction was defined according to current guidelines.^7,8^

The study complies with the Declaration of Helsinki and good clinical practice guidelines and was approved by the responsible local ethics committee (project number 076/2021B02). The study follows the Strengthening the Reporting of Observational Studies in Epidemiology reporting guidelines.

### Simple logistic regression models and supervised machine learning models

For evaluating the predictive value of the key diagnostic points [symptoms, risk factors, ECG, echocardiography, troponin, and coronary angiography (CA)], logistic regression models were trained in R using the packages ROCit (2.1.1), precrec (0.14.4), FSA (0.9.5), and caret (6.0–49) and Python using scikit-learn (1.5.2). A 75/25% split was used for training and validation. Logistic regression models were both applied to single diagnostic ladder steps and the combination of previous ladder steps, by using all available variables up to that point. The same variables were used for the XGBoost models later. To explore interactions between variables, differences in prediction using the ‘+’ and ‘*’ operator within caret were compared for two variables using the χ^2^ test. Cross-fold validation was used here.

The XGBoost models were implemented by scikit-learn (1.5.2). In detail, we set up different XGBoost models for binary classification of revascularization need or not. Prior to ML model training, patients with more than 20% of missing values were filtered from the dataset, and remaining missing values were imputed by mean for continuous data, median for ordinal data, and modus for categorical data for training and validation datasets. Percentages of missing values are shown in Supplementary material online, *Table S2*. Next, features were scaled using scikit-learn’s StandardScaler for continuous data or MinMaxScaler for ordinal data. Following standard ML methodology, the dataset was randomly split into a training (75% of cases) and validation (25% of cases) dataset to perform a nested cross-fold validation approach. Within the nested training loop, the binary classifiers were trained including hyperparameter tuning and 10-fold cross-validation. Subsequently, the trained classifiers were applied to previously unseen data of the validation dataset (25% of population) to avoid overfitting. To rule out the influence of different patient compositions, group sizes, or imputations on performance of the models, a sub-cohort-analysis with intersecting patients from all four models and without imputation was performed (see Supplementary material online, *Figure S6*).

Variables included in the ML models were chosen based on clinical relevance, availability at defined diagnostic time points, and prior evidence of association with coronary artery disease (CAD). The four XGBoost models were built incrementally to reflect real-world clinical decision-making, taking into consideration all clinically relevant and available variables at the specific diagnostic time point.

### Unsupervised machine learning

For phenotypization of the patient cohort, unsupervised analyses were conducted on the patient cohorts of each individual XGBoost model. Uniform Manifold Approximation and Projection (UMAP) was used for dimension reduction with Gower’s distance as the distance metric, in order to calculate similarity and dissimilarity of the variables’ mixed data types, as also described previously.^9,10^

### Misclassification analysis

To identify patients susceptible to misclassification, analysis on variables contributing to over- or underprediction of coronary revascularization need was performed. The background idea is that a ML model uses the combination of all variables and knowledge on the training dataset to predict a pre-specified outcome for patients in the validation dataset. Misclassified patients were those where the predicted probability deviated >0.75 from the actual outcome. In detail, coronary revascularization need was coded binary as 0 if false and 1 if true. Consecutively, overpredicted patients were those where the ground truth was 0 but the predicted probability was larger than 0.75. Vice versa, underpredicted patients were those with a ground truth of 1 and a predicted probability lower than 0.25. Misclassification of the best performing Models 3 and 4 was considered.

### Decision curve analysis

To assess the clinical applicability of the ML models, decision curve analyses were performed. Here, the risk threshold is plotted against the net benefit of a model. Net benefit (treated) is defined as the number of true positive patients minus the weighted number of false positive patients, divided by the total number of patients. The R package rmda (version 1.6) was used for analysis.

### Statistical analysis and visualization

Data were analysed using SPSS 28.0.0.0, Python 3.10.12, and R 4.3.0, each applying packages as described before. The model’s classification performance was in Supplementary material online, *Table S1*. Shapiro–Wilk tests showed that clinical parameters were not normally distributed. Metric data are reported as median and interquartile range (IQR). Categorical data are given as frequencies and percentages. χ^2^ test was used to compare categorical variables, whereas the Mann–Whitney *U* test/Kruskal–Wallis test and following Dunn’s test (two groups) or Wilcox test (more than two groups) was used to compare metric variables.

For visualization of ROC curves, we used Python’s matplotlib (3.7.1). The package shap (0.43.0) was used to visualize SHapley Additive exPlanations (SHAP) values and provide explainability to our classifier models. For circular Sankey plots, the R package circlize (0.4.1) was applied.

## Results

**Figure 1:**
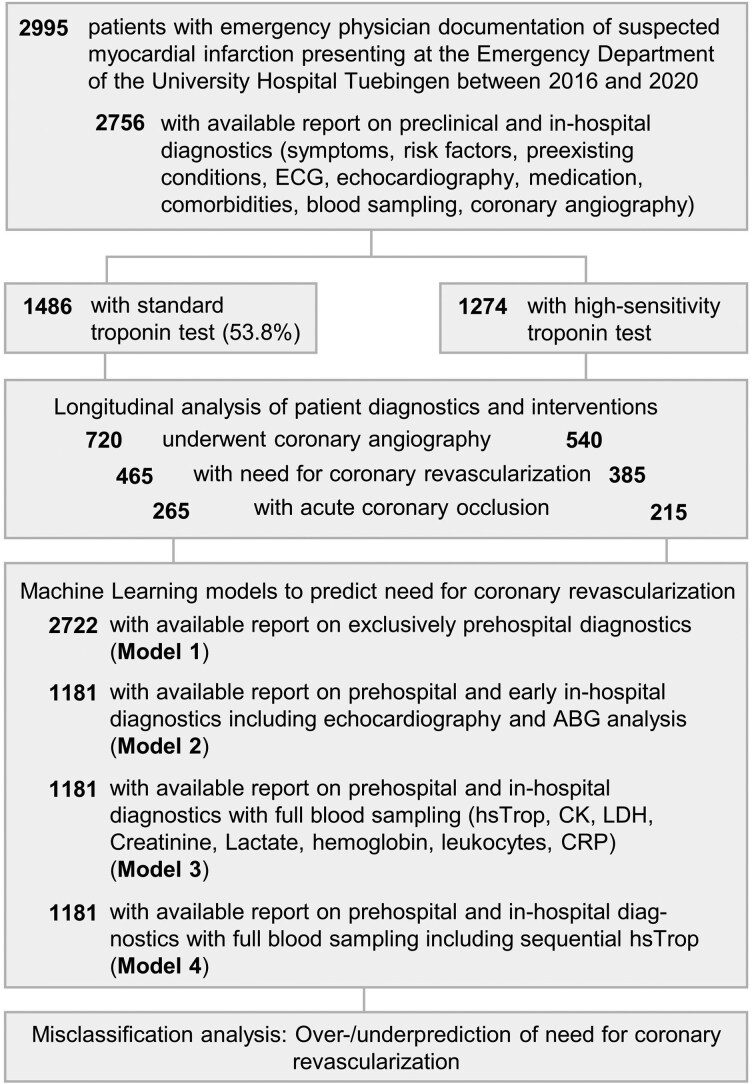
Study overview. Inclusion of patients, relevant prehospital and in-hospital variables, number of standard troponin vs. high-sensitive troponin testing. Longitudinal patient trajectories starting from prehospital presentation until coronary angiography were then investigated.

A total of 2756 patients between 2016 and 2020 with longitudinal pre- and in-hospital diagnostic data were included (*Figure 1*). A total of 53.8% received standard Trop testing and 46.2% high-sensitivity troponin (hsTrop) testing at the hospital. Four ML models based on eXtreme Gradient Boosting (XGB) were trained on data available at different diagnostic time points, ranging from exclusive prehospital assessments to advanced in-hospital diagnostics. Models were trained to predict the need for coronary revascularization and internally validated on hold-out data of the patient dataset. Coronary revascularization need was defined as coronary angiography with percutaneous transluminal coronary angioplasty (PTA) and/or coronary intervention (PCI), including cases where coronary angiography showed severe stenosis with the need for or early bypass operation within 48 h after hospital arrival.

Baseline characteristics (*Table 1*) showed that patients in need for coronary intervention were more often male, complained of stronger pain, and were most frequently classified as ‘STEMI’ by the treating EP due to more prevalent ST-elevations. Furthermore, patients in need of coronary intervention had higher concentrations of haemoglobin (Hb), lactate dehydrogenase (LDH), creatinine kinase (CK), C-reactive protein (CRP), lactate, and creatinine but demonstrated lower leucocyte counts. Cardiovascular risk factors such as hypertension, CAD, diabetes, and nicotine consumption were also more prevalent. Lastly, they had more regional wall motion abnormalities (RWMAs) and impaired left ventricular ejection fraction (LVEF) on echo than in patients without intervention need.

**Table 1:** Patient characteristics including details on prehospital presentation and in-hospital diagnostics

Parameter	All patients, *n* = 2756	Coronary revascularization, *n* = 850 (30.8%)	*P*-value
*Clinical data*			
Age (years)	70 (23)	70 (19)	0.075
Gender (male)	1615 (58.6)	591 (69.5)	**<0**.**001**
*Preclinical diagnoses*			
STEMI	362 (13)	252 (69.6)	**<0**.**001**
NSTEMI	2024 (73,4)	559 (27.6)	**<0**.**001**
Chest pain	370 (13.4)	39 (10.5)	**<0**.**001**
*Preclinical data*			
ST-elevation	422 (18)	267 (35.4)	**<0**.**001**
ST-depression	205 (8.8)	78 (10.3)	0.060
Heart rate	82 (28)	81 (25)	0.970
Blood pressure	150 (40)	150 (40)	0.602
SpO_2_	97 (3)	97 (3)	0.662
Pain intensity	4 (5)	4 (5)	**0**.**019**
Symptom onset (minutes)	150 (363)	106 (389)	0.277
*Laboratory results*			
BNP (ng/L)	1696 (6730)	1677 (7881)	0.437
Hb	13,2 (2.5)	13,5 (2.6)	**<0**.**001**
LDH	191 (70)	206 (103)	**<0**.**001**
Initial Trop (µg/L)	0.1 (0.63)	0.28 (1.91)	**<0**.**001**
Initial hsTrop (µg/L)	11 (54)	84 (1082)	**<0**.**001**
AllTrop+	985 (35.7)	601 (70.7)	**<0**.**001**
Creatinine kinase	104 (131)	209 (625)	**<0**.**001**
CRP (mg/dL)	0.27 (0.97)	0.35 (1.16)	**<0**.**001**
Leucocytes	8360 (4070)	7960 (4735)	**<0**.**001**
Lactate	1.5 (0.9)	1.7 (1.2)	**<0**.**001**
Creatinine	0.9 (0.4)	0.9 (0.3)	**<0**.**001**
*Patient history*			
Hypertension	2098 (76.4)	724 (85.2)	**<0**.**001**
Diabetes	680 (24.8)	260 (30.6)	**<0**.**001**
Known CAD	1146 (41.8)	408 (48)	**<0**.**001**
Nicotine	562 (20.4)	210 (24.7)	**<0**.**001**
BMI	26.9 (6.9)	27 (6.2)	0.386
*Echocardiographic findings*			
RWMA	634 (25.5)	404 (50.2)	**<0**.**001**
LVEF impaired	820 (32.6)	458 (55.8)	**<0**.**001**
*Coronary angiography findings*			
No. of vessels affected	3 (1)	3 (1)	**<0**.**001**

Bold *P*-values highlight significant differences. Continuous variables are expressed as medians with IQR; dichotomous variables are presented as frequency with percentages. Mann–Whitney *U* tests were used for metric variables whereas χ^2^ tests were used to compare dichotomous variables. Pain intensity is rated on a numeric scale ranging from 0 (for no pain) to 10 (the worst pain).

RWMA, regional wall motion abnormality; STEMI, ST-elevation myocardial infarction; NSTEMI, non-ST-elevation myocardial infarction; CK, creatinine kinase; LDH, lactate dehydrogenase; CRP, C-reactive protein; Hb, haemoglobin; BNP, brain natriuretic peptide; LVEF, left ventricular ejection fraction; CAD, coronary artery disease; BMI, body mass index.

### Diagnostic ladder to coronary vascular diagnosis

**Figure 2:**
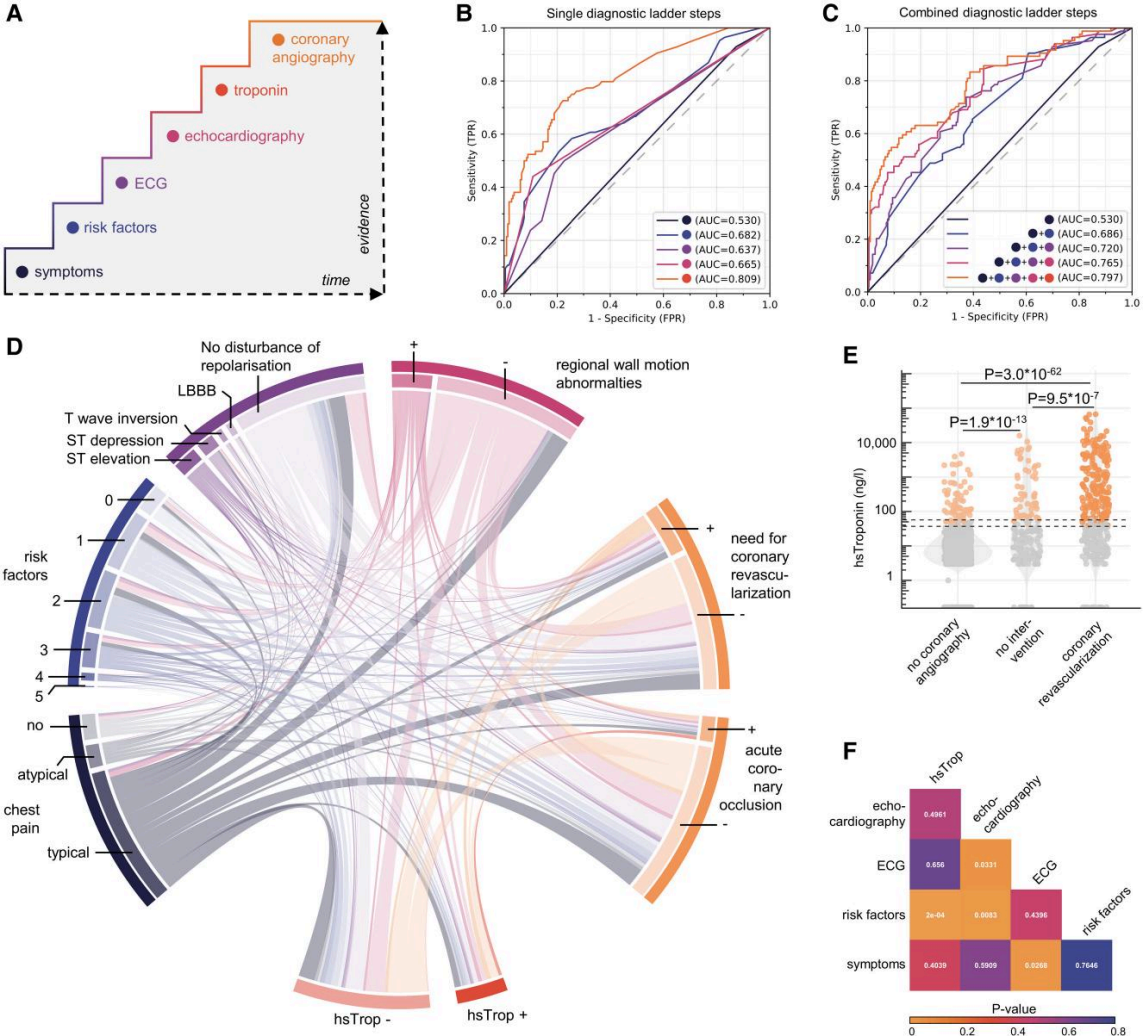
Diagnostic path to coronary vascular diagnosis. (*A*) Diagnostic ladder describing an increasing level of probability in dependence of additional diagnostics, which require time from symptom onset. (*B*) ROC curve visualizing sensitivity and specificity of single diagnostic items from the diagnostic ladder to predict the need for coronary revascularization. Logistic regression was used as the model. Dotted line represents models including standard troponin testing in contrast to high-sensitivity troponin with a solid line. (*C*) Prediction of the need for coronary revascularization by averaging the predicted probabilities from items of the diagnostic ladder. Logistic regression. (*D*) Relationship network visualized in a circular Sankey plot focused on diagnostic value of high-sensitivity troponin. The width represents the absolute frequency of each item. Edges are bidirectional and link each item with each other item. (*E*) Quantification of diagnostic value of high-sensitivity troponin for estimating the need for coronary revascularization. The dotted lines represent the cut-offs for elevated troponin levels, which are sex dependent in case of high-sensitivity troponin. Kruskal–Wallis test Dunn’s test with Holm adjustment for multiple testing. (*F*) Heatmap displaying interactions between single steps of the diagnostic ladder. Interaction terms were measured from logistic regression models and compared with the respective non-interacting variables by χ^2^ test. *P*-values are color-coded. Trop, troponin; hsTrop, high-sensitivity troponin; ROC, receiver operating characteristic curve; FPR, false positive rate; TRP, true positive rate.

Acute coronary syndrome diagnosis follows a stepwise approach, where each diagnostic step increases evidence for intervention need but requires more time (*Figure 2A*). Patients first undergo prehospital assessment based on symptoms, risk factors, and ECG. Upon hospital arrival, echocardiography and blood tests follow. ST-elevation on prehospital ECG leads to direct catheter lab transfer, where quick echocardiography is performed on the catheter table and blood is drawn periinterventionally.

Considering the diagnostic value of individual ladder items, early markers provided limited predictive value, while vice versa, later available diagnostics such as echocardiography and sequential Trop testing demonstrate high sensitivity and specificity but delay the decision to perform CA. Logistic regression models were used for basic benchmarking, showing AUC values of 0.540 for typical symptoms alone, 0.619 for presence of risk factors, and 0.642 for ACS-relevant depolarization disorders (ST-elevations, ST-depressions, T-wave inversions, and LBBBs) (*Figure 2B*). High-sensitivity troponin testing yielded an AUC of 0.790.

We then investigated whether combining all available data in a stepwise process could accelerate decision-making for coronary angiography while minimizing the risk of coronary angiography without the need for revascularization. We observed that especially combination of early available variables such as symptoms, risk factors, and ECG critically enhanced sensitivity and specificity (AUC 0.706), compared with the single diagnostic available at that time point (AUC 0.54–0.642) (see *Figure 2C* and Supplementary material online, *Figure S1A*). This was also the case, but less significant, for the later-stage logistic models (maximum AUC 0.797).

A special case of need for coronary revascularization is acute coronary occlusion, defined as coronary stenosis of >99%. As typical patients have more severe symptoms, depolarization abnormalities, and higher Trop levels, acute coronary occlusion prediction performed slightly better than overall coronary revascularization need (maximum AUC 0.853; see Supplementary material online, *Figure S1B* and *C*).

### Patient trajectories from prehospital presentation to in-hospital diagnosis

We analysed how patients with suspected ACS longitudinally follow the diagnostic pathway. Therefore, we investigated the patient’s diagnostic ‘journey’ in a relationship network (*Figure 2D*). When looking at the cohort of patients with elevated initial Trop or hsTrop levels, a considerable fraction of patients received coronary angiography without revascularization need. Conversely, almost no patients had acute coronary occlusion without elevated hsTrop. This highlights the fact that even though sensitive for myocardial cell necrosis, Trop is not a specific marker for the origin of cardiac cell death (i.e. severe coronary artery stenosis).

Further stratification showed that Trop levels were significantly higher in those requiring revascularization or with coronary occlusion (*Figure 2E*; Supplementary material online, *Figure S3*), but also elevated in many non-intervention cases. This pattern was also replicable in patients where standard Trop testing was performed (see Supplementary material online, *Figure S3*), emphasizing the need to critically question isolated Trop elevations. To investigate the relationship between Trop levels and predicted outcomes, we used restricted cubic splines. Notably, spline fits showed an earlier increase in predicted probabilities at lower concentrations for hsTrop compared with conventional Trop (see Supplementary material online, *Figure S4*). This suggests superiority in risk discrimination, particularly for lower hsTrop values, compared with conventional Trop.

Logistic regression is well known for providing stable and interpretable estimates for individual predictors, but it requires explicit modelling of interaction terms and may not adequately capture complex, non-linear relationships—especially when interactions are not pre-specified. To explore whether such interactions exist between components of the diagnostic ladder, we tested interaction terms in logistic regression models (*Figure 2F*). Compared with their isolated consideration, significant interactions between ECG and echocardiographic wall motion abnormalities (*P* = 0.0331), as well as between symptoms and ECG (ST-elevation or ST-depression, *P* = 0.0268) were found. The most notable interactions were observed between risk factors and Trop levels (*P* = 2 * 10^−4^) and between risk factors and echocardiographic wall motion abnormalities (*P* = 0.0083).

These findings suggest that modelling strategies capable of automatically detecting complex interactions may offer diagnostic advantages. Accordingly, we applied XGBoost, a non-linear, tree-based ML model, to the diagnostic steps. While logistic regression performed slightly better in modelling isolated diagnostic steps, XGBoost demonstrated superior performance when multiple diagnostic components were combined (see Supplementary material online, *Figure S2*). Based on this, we proceeded with XGBoost as our primary modelling approach for Models 1–4.

### Establishment of sequential machine learning models to predict the need for coronary revascularization

**Figure 3:**
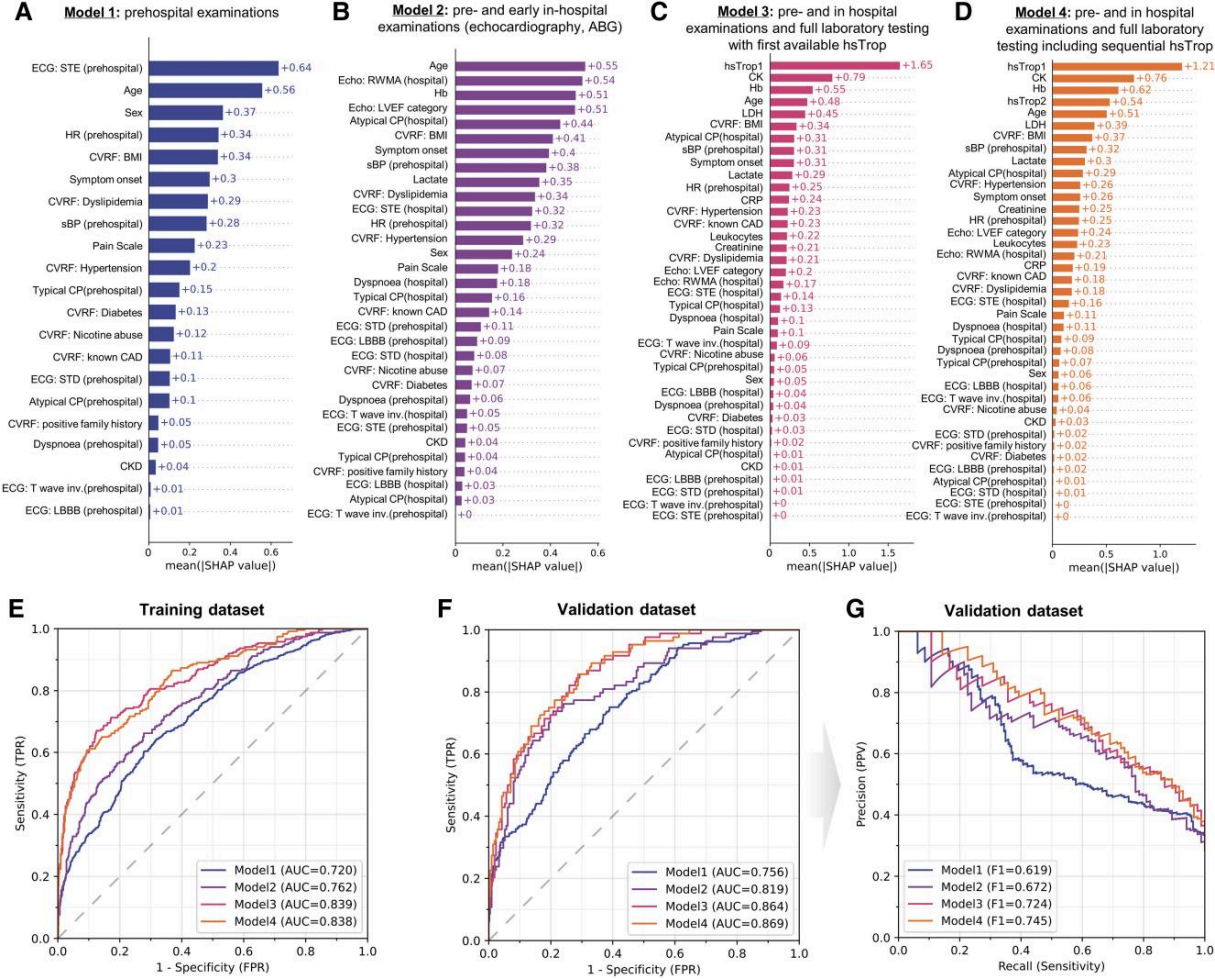
Establishment of machine learning models to predict the need for coronary revascularization. (*A–D*) Mean absolute SHapley Additive exPlanations values for the different models. Bar plots are sorted by the highest SHapley Additive exPlanations value. (*E*) Receiver operating characteristic curve curve for the training dataset (75% of data) using 10-fold cross-validation. (*F*) Receiver operating characteristic curve curve for validation dataset (25% of data). (G) Corresponding precision-recall curve for the validation dataset. SHAP, SHapley Additive exPlanations; ROC, receiver operating characteristic curve

To overcome the previously described limitations and allow the highest predictions for coronary revascularization need at the earliest time possible, we trained four different ML models, each of which incorporated patient data available at different diagnostic time points (*Figure 3*). Model 1 used prehospital data only, Model 2 incorporated prehospital data, but also follow-up analysis of symptoms and ECG, as well as echocardiography and results from ABG. All these diagnostics are available within 15 min after arrival at the ED. Model 3 added full laboratory testing, requiring another 45 min for all relevant parameters. This model includes the first available hsTrop value. Model 4 adds to Model 3 a sequential hsTrop testing after another 1–2 h.

Unsurprisingly, our models demonstrate increasing predictive value with the addition of more variables (see *Figure 3E–G* and Supplementary material online, *Table S1*), but also superiority towards simple logistic regression models from before (maximum AUC 0.869 vs. 0.797) (*Figure 2C*). Comparing the performance of XGBoost and logistic regression across Models 1–4 revealed that XGBoost showed a slight but consistent advantage, particularly in terms of AUC, AUPRC, and calibration (see Supplementary material online, *Table S1* and *Figure S7*). The frequency distribution of predicted probabilities can be found in Supplementary material online, *Figure S1C*. To rule out diagnostic performance variations due to differences in patient cohort compositions, we analysed the intersecting patient sub-cohort that was used throughout all models (1172 patients). Notably, using this reduced cohort in Model 1 led to a notably lower AUC (0.650 vs. 0.720 in training; 0.668 vs. 0.756 in validation), whereas Models 3 and 4 showed minimal differences (Model 3: 0.839 vs. 0.839 in training, 0.835 vs. 0.864 in validation; Model 4: 0.832 vs. 0.838 in training, 0.863 vs. 0.869 in validation; see Supplementary material online, *Figure S6*). Calibration analysis showed good agreement between predicted probabilities and the actual need for revascularization, with the lowest Brier scores observed for Model 4 of 0.144 in the full cohort and 0.120 in the intersecting sub-cohort (see Supplementary material online, *Figure S7*).

To improve model explainability, SHAP analysis was performed for each model (*Figure 3 A–D*). Prehospital ECGs, particularly ST-elevations, were most critical in Model 1 (+0.64) but less so in Model 2 (+0.05), where echocardiographic RWMA gained importance at this stage (+0.55) (*Figure 3A* and *B*). Troponin levels dominated Models 3 (+1.65) and 4 (+1.21), but sequential measurements were less impactful (+0.54) than initial values (*Figure 3C* and *D*).

To further evaluate the performance of our XGBoost models, we compared them against established clinical scoring systems. While no existing score has been specifically developed to predict the need for coronary revascularization, the preHeart,^11^ HEART,^12^ and mini-GRACE^13^ score may serve as surrogate benchmarks, although focusing mainly on prediction of major adverse cardiac events. Using the Net Reclassification Index (NRI) to assess relative performance, all XGBoost models demonstrated superior reclassification ability compared with these conventional scores, with the exception of Model 1 vs. the HEART score, where performance was comparable (see Supplementary material online, *Figure S8*). However, XGBoost Model 1 predictions are already available prehospitally, offering a substantial time advantage compared with the HEART score requiring laboratory Trop testing.

To explore if unsupervised ML can guide phenotypization of the patient cohort, we used UMAP with Gower’s distance for dimension reduction and k-medoids clustering to stratify patients into phenotypic groups based on the variables used for training the XGBoost Models 1–4. However, meaningful subgroup separation was not achieved (see Supplementary material online, *Figure S9*), highlighting the importance of supervised learning for identifying patients requiring coronary revascularization.

### Misclassification analysis

Despite optimizations of the XGB-based models, some misclassification persisted. Overpredicted patients were defined as those with high model-predicted probabilities (>0.75) but no revascularization in ground truth, while underdiagnosed patients had revascularization need but model probabilities of <0.25 (see Supplementary material online, *Figures S5A* and *S3B*). By statistical comparison of variables between misclassified and correctly classified patients, we aimed to identify potential predictors for misclassification.

**Figure 4:**
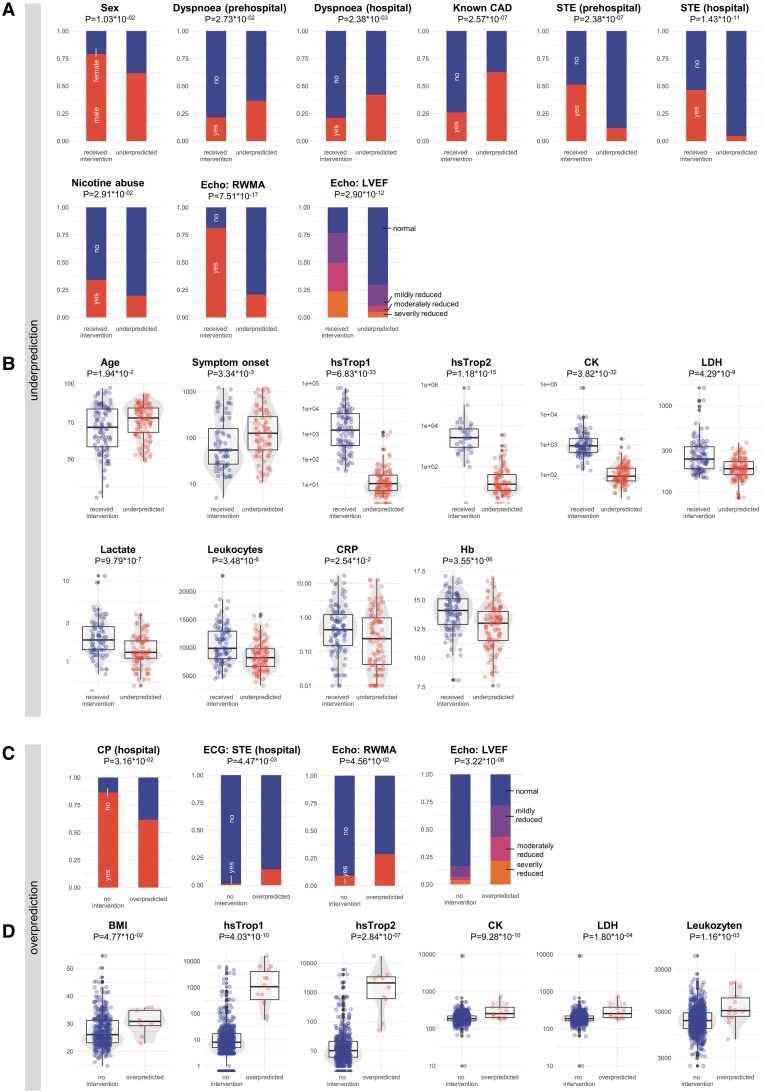
Misclassification analysis. (*A* and *B*) Quantitative analysis of variables significantly different in patients with underpredicted coronary revascularization need. Binary variables in the top row (*B*) and continuous variables in the bottom row (*B*). (*C* and *D*) Quantitative analysis of variables significantly different in patients with overpredicted need of coronary revascularization. Binary variables in the top row (*B*) and continuous variables in the bottom row. Wilcox test for continuous data, Kruskal–Wallis test for ordinal data, and χ^2^ test for binary data.

Underdiagnosed patients were often older females with persistent dyspnoea during prehospital and in-hospital presentation. Patients had more often previously known CAD, but did not show ST-elevations at the EP’s ECG or at the hospital. Notably, regional wall motion abnormalities and impaired LVEF were significantly less observed in these patients, and laboratory values such as hsTrop, creatinine kinase (CK), lactate dehydrogenase (LDH), C-reactive protein (CRP), haemoglobin (Hb), and inflammatory markers were lower (*Figure 4A* and *B*). Sequential Trop changes were observed in both correctly and underpredicted patients, indicating that while the delta was statistically significant, it added little incremental predictive value beyond the baseline hsTrop measurement (see Supplementary material online, *Figure S10A*). Specifically, underpredicted patients tended to have lower initial hsTrop levels, suggesting that baseline elevation, rather than the absolute change, had greater influence on model prediction.

Overdiagnosed patients exhibited multiple high-risk features, including ST-elevations, RWMA, and elevated biomarkers, yet did not require intervention (*Figure 4C* and *D*). Some cases may have involved myocarditis or aortic dissection. In these cases, lack of sequential Trop rise could have helped identify these patients (see Supplementary material online, *Figure S10B*). However, additional Trop testing may critically delay activation of the catheter laboratory in this high-risk cohort.

### Clinical decision-making

**Figure 5:**
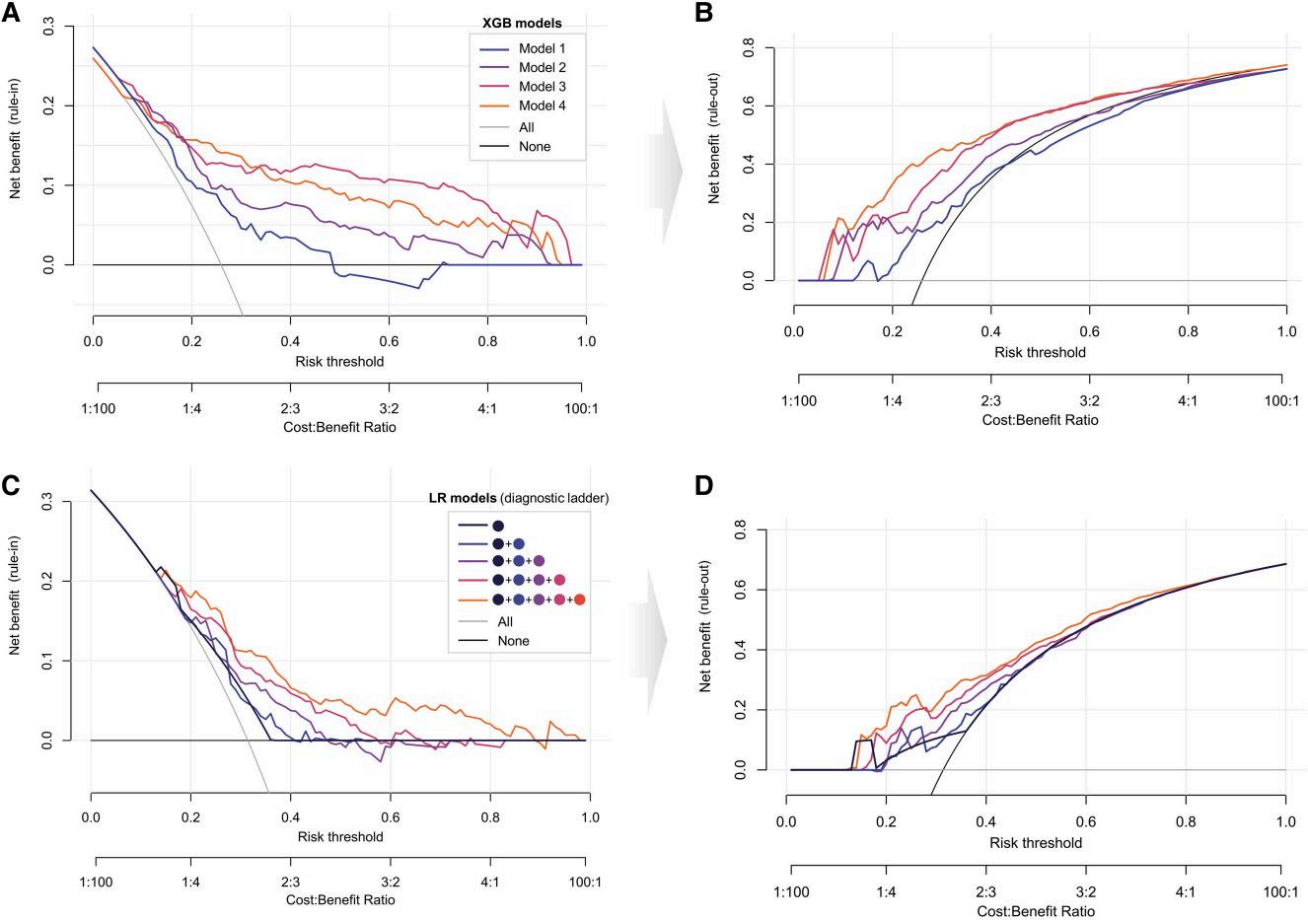
Decision curve analysis for XGBoost models and diagnostic ladder steps. (*A* and *B*) Decision curve analysis of XGBoost Models 1–4 on the identical sub-cohort. showing the net benefit across a range of risk thresholds for clinical decision-making. The ‘None’ line (black) reflects the strategy of subjecting no patients to coronary angiography, while the ‘All’ line (grey) represents performing coronary angiography on all patients. Among the models, for rule-in (*A*) of patients at intermediate- to high-risk thresholds, Model 3 (pink) shows the highest net benefit, indicating superior decision-making utility in this setting. For lower risk thresholds, rule-out analysis (*B*) showed the best performance of Model 1 (orange) in avoiding unnecessary coronary interventions. (*B* and *C*) Decision curve analysis of logistic regression models applied to the diagnostic ladder and subsetted by the identical sub-cohort, differentiating between rule-in (*C*) and rule-out (*D*) strategies. XGB, XGBoost; LR, logistic regression.

To assess the clinical applicability of our models and their potential to guide decision-making regarding the need for coronary angiography, we performed a decision curve analysis (*Figure 5*). This method evaluates the net benefit of each model across a range of clinically relevant risk thresholds by balancing the true positive rate against the weighted consequences of false positives. To rule out the influence of different patient cohort compositions, we used the same intersecting sub-cohort as described before.

When analysing the identical patient cohort by the XGBoost models (*Figure 5A*), Model 3 demonstrated the highest net benefit across the widest span of thresholds, even outperforming the later-stage Model 4, that incorporates sequential Trop testing. This suggests that Model 3 offers the highest utility for guiding revascularization decisions (‘rule-in’) in patients with suspected ACS. Notably, even at very high probability thresholds, Model 3 offered greater benefit over other models and a conservative ‘treat-none’ strategy. This indicates that waiting for sequential Trop testing in patients with intermediate–high-risk threshold may not provide additional benefit.

For lower risk thresholds, the ‘rule-out’ analysis (*Figure 5B*) illustrates how the models can effectively reduce the number of interventions that can be avoided. Here the net benefit corresponds to the net reduction in unnecessary coronary interventions. The late stage Model 4 performed best here, achieving a net reduction of around 0.3 at a risk threshold of 0.2, compared with performing coronary angiography on all patients. At lower risk thresholds, the net benefit between the models were less pronounced, suggesting that early, data-sparse predictions can still provide meaningful support, particularly when the clinical goal is to identify low-risk patients who may not require immediate invasive diagnostics.

The similar analysis was repeated for the logistic regression models of the diagnostic ladder (*Figure 5C* and *D*). The decision curves indicated that incorporating the maximum available evidence yields the best performance for both ‘rule-out’ and ‘rule-in’ strategies. Importantly, net benefits were markedly higher with the advanced XGBoost models, supporting their utility and clinical usefulness in guiding treatment decisions.

## Discussion

In our study, ML methodology was used to predict the need for coronary revascularization in patients with suspected ACS. In contrast to other prehospital studies,^10,14,15^ this is the first study that uses a longitudinal approach and integrates pre- and in-hospital data at different time points of the diagnostic process. This way, we were not only able to predict the endpoint but also shine light on the importance of specific diagnostic steps and their impact on model performance.

Given the time-sensitive nature of ACS, referral for catheterization often occurs before a full diagnostic workup. However, overdiagnosis remains a concern, as coronary angiography is an invasive procedure that can be associated with adverse outcomes, complications, and exposure to radiation.

We observed that combination of diagnostic modalities critically improved diagnostic performance, especially at early diagnostic time points. While logistic regression, used as a basic benchmarking model, was limited to evaluating one variable at a time or averaging probabilities across steps, XGBoost was better suited for our goal of integrating information across multiple diagnostic stages due to its ability to capture complex interactions between variables. Even though hsTrop, which is considered standard of care and recommended in current guidelines,^8,16^ showed the highest individual area under the receiver operating characteristic (AUROC) for the need for coronary revascularization, integration of patient history, ECG, and echocardiography provided comparable accuracy with faster availability. We observed that predictive models for acute coronary occlusion outperformed those for general coronary revascularization need, likely due to the more pronounced clinical and diagnostic markers in occlusion cases. Machine learning algorithms to identify this patient cohort have been used successfully before.^17^

To improve predictive power, multiple XGboost-based ML algorithms were trained to predict the need for coronary revascularization on a longitudinal approach. Since obtaining valid diagnostic requires time, clinicians have to leverage diagnostic accuracy to potential harm in delaying therapy. Our models can help getting an evidence-based prediction for need of coronary revascularization with estimation of sensitivity and specificity at multiple time points. For each time point, SHAP analysis helped identify the most important parameters for risk stratification. Overall, SHAP analysis highlighted the importance of careful prehospital ECG interpretation and point-of-care echocardiography.^18,19^ Although Trop was a major predictor, sequential measurements had less impact than initial values, likely reflecting symptom onset times. Median symptom-to-assessment time was 150 min in our cohort, with further delays for blood tests, suggesting initial Trop levels might have already sufficiently reflected onset of myocardial damage in most patients.^20^

Even though the use of cardiac Trop is a cornerstone of modern cardiology and emergency medicine and the best singular marker in our models, the parameter is not specific for myocardial cell necrosis due to CAD.^21^ As mentioned in the Results section, multiple patients showed elevated Trop levels but had no need for revascularization. Even though Trop was significantly higher in patients receiving coronary revascularization or suffering from acute coronary artery occlusion, some levels were above the clinical cut-off values^22–24^ in non-occlusion/non-revascularization patients as well. This further highlights the need for integrating multiple clinical variables besides Trop to avoid overdiagnosis.

Misclassification analysis revealed that some high-risk patients, such as those with ST-elevations, RWMA, and impaired LVEF, did not require revascularization. Conditions such as aortic dissection, myocarditis, or Takotsubo cardiomyopathy may explain these cases. Additional factors such as infection history presence of neurologic symptoms or blood pressure difference between extremities might help in early identification of these outliers.^25^ Additionally, lack of increase in sequential Trop testing might also point towards a non-coronary cause. However, when in doubt, diagnostic coronary angiography in those high-risk individuals is still warranted.

Underdiagnosed patients were often older females with dyspnoea, absent ST-elevations, and lower cardiac biomarker levels. Interestingly, this reflects the clinical reality where CAD in female patients is still underrecognized due to ‘atypical’ presentation.^26–28^ While some of these patients may not have required urgent revascularization, clinicians should remain vigilant to avoid missed diagnoses in this subgroup.

We are aware that our endpoint depends on the clinical judgment of the treating cardiologist. However, unselective revascularization is not indicated in all patients with myocardial infarction. Coronary revascularization should not be performed in patients with Type-II myocardial infarction, and recent studies have challenged the effectiveness of the procedure in older, frail patients.^29^ Conversely, CAD with certain anatomical features requires revascularization in the absence of acute myocardial cell loss.^30^ We therefore felt that in the absence of follow-up data on mortality, the need for revascularization is a clinically relevant endpoint.

In clinical practice, our model could be used to track the need for coronary revascularization in an evidence-based way along the diagnostic pathway with increasing precision and recall. This could potentially reduce overdiagnosis and help identify patients with the need for coronary revascularization at earlier time points.

## Study limitations

Since not all patients with suspected ACS received coronary angiography, we cannot exclude missing patients in need for revascularization. However, it is good clinical practice to impose strict indications for the procedure since coronary angiography can lead to adverse events and radiation exposure. Additionally, our prehospital model may not directly generalize to all healthcare systems, as EPs—not paramedics—performed initial evaluations, potentially affecting ECG interpretation quality due to differences in training.

Another limitation of our study is the lack of external validation. While our models demonstrated robust performance in internal cross-validation and on a separate hold-out validation cohort, their generalizability to other populations and clinical settings remains unproven. External validation using independent datasets from different healthcare systems or regions is essential to confirm the model’s reliability and transportability. Future studies should aim to validate these findings externally to ensure the applicability of our approach in broader clinical practice.

## Conclusions

Our models could help clinicians evaluate patients with suspected ACS and may reduce overdiagnosis. Owing to the longitudinal approach, patients in need for coronary revascularization can be identified at different time points with increasing precision and recall. Furthermore, we were able to identify the most important clinical variables for successful prediction of coronary revascularization and identified patient subpopulations at risk for over- and underdiagnosis. This might help to improve patient outcomes in individuals with suspected ACS.

## Supplementary Material

ztaf106_Supplementary_Data

## Data Availability

Python and R code for our ML model is available from the corresponding author upon reasonable request. Any commercial use (that includes sale, license, distribution, lease, or transfer) is prohibited.

## Author statement

This author takes responsibility for all aspects of reliability and freedom from bias of the data presented and their discussed interpretation.
