# Supplementary material for: AI-guided refinement of coronary revascularization need in patients suspected of acute coronary syndrome
Source: Eur Heart J Digit Health. 2025 Oct 6;6(6):1169–80. doi: 10.1093/ehjdh/ztaf106 (PMC12629660; doi:10.1093/ehjdh/ztaf106)
Supplement: ztaf106_Supplementary_Data [file ztaf106_supplementary_data.pdf]

Supplementary material online to

AI-guided refinement of coronary revascularization need in patients suspected of acute coronary syndrome

Running title: AI-guided reduction of unnecessary coronary angiography in patients suspected of ACS

Manuel Sigle, MD, Diana Heurich, MD, Wenke Faller, MD, Meinrad Gawaz, MD, PhD, Karin Anne Lydia Mueller, MD*, Andreas Goldschmied, MD

Department of Cardiology and Angiology, University Hospital Tübingen, Eberhard Karls University Tübingen, Tübingen, Germany

***Corresponding author:**

Karin Anne Lydia Mueller

Department of Cardiology and Angiology, University Hospital Tübingen, Eberhard Karls University Tübingen

Otfried-Müller-Str. 10, 72076 Tübingen, Germany

Email: K.Mueller@med.uni-tuebingen.de

Supplemental Tables

Table S1. Evaluation of model classification performance (validation dataset).

		AUC	Accuracy	Precision (weighted)	Recall (weighted)	Specificity	F1 Score (weighted)	AUPRC
**XGBoost**	**Model 1**	0.756 (0.719-0.792)	0.652 (0.616-0.688)	0.794 (0.782-0.807)	0.652 (0.616-0.688)	0.611 (0.566-0.655)	0.687 (0.654-0.72)	0.619 (0.555-0.682)
	**Model 2**	0.819 (0.765-0.872)	0.775 (0.727-0.822)	0.778 (0.728-0.827)	0.775 (0.727-0.822)	0.793 (0.738-0.849)	0.786 (0.741-0.831)	0.672 (0.572-0.772)
	**Model 3**	0.864 (0.823-0.906)	0.747 (0.697-0.797)	0.815 (0.797-0.832)	0.747 (0.697-0.797)	0.708 (0.647-0.768)	0.778 (0.736-0.82)	0.724 (0.63-0.817)
	**Model 4**	0.869 (0.827-0.911)	0.749 (0.698-0.800)	0.817 (0.800-0.835)	0.749 (0.698-0.8)	0.712 (0.65-0.774)	0.779 (0.734-0.824)	0.745 (0.657-0.834)
**Logistic regress.**	**Model 1**	0.760 (0.722-0.797)	0.758 (0.725-0.791)	0.747 (0.711-0.782)	0.758 (0.725-0.791)	0.879 (0.849-0.91)	0.752 (0.718-0.786)	0.628 (0.559-0.696)
	**Model 2**	0.797 (0.74-0.854)	0.683 (0.633-0.734)	0.782 (0.738-0.827)	0.683 (0.633-0.734)	0.623 (0.561-0.685)	0.729 (0.686-0.773)	0.671 (0.577-0.766)
	**Model 3**	0.814 (0.759-0.869)	0.777 (0.731-0.824)	0.786 (0.739-0.833)	0.777 (0.731-0.824)	0.826 (0.776-0.877)	0.782 (0.736-0.828)	0.708 (0.618-0.798)
	**Model 4**	0.814 (0.76-0.868)	0.778 (0.732-0.824)	0.787 (0.741-0.833)	0.778 (0.732-0.824)	0.826 (0.774-0.877)	0.782 (0.737-0.828)	0.704 (0.617-0.791)

mean, 95% confidence interval
AUC – area under the curve
AUPRC – area under the precision-recall-curve

Table S2. Percentage of missing values for each model's dataset

Model 1	missing values (%)
BMI	42.359
ECG: STE (prehospital)	14.732
ECG: STD (prehospital)	14.732
ECG: T wave inv.(prehospital)	14.732
ECG: LBBB (prehospital)	14.732
Symptom onset	14.622
Typical CP (prehospital)	9.295
HR (prehospital)	1.396
SBP (prehospital)	1.065
Dyspnoea (prehospital)	0.184
Typical CP (prehospital)	0.147
Atypical CP (prehospital)	0.147
CVRF: Diabetes	0.073
CVRF: known CAD	0.037
CKD	0.037
CVRF: positive family history	0.000
CVRF: Nicotine abuse	0.000
CVRF: Hypertension	0.000
CVRF: Dyslipidemia	0.000
Sex	0.000
Age	0.000

Model 2	missing values (%)
BMI	40.644
ECG: STE (prehospital)	29.721
ECG: STD (prehospital)	29.721
ECG: T wave inv.(prehospital)	29.721
ECG: LBBB (prehospital)	29.721
Lactate	22.608
Symptom onset	21.423
Typical CP (prehospital)	12.616
Echo: RWMA	8.806
Echo: LVEF category	7.875
ECG: LBBB (hospital)	2.625
ECG: STD (hospital)	2.625
ECG: STE (hospital)	2.625
ECG: T wave inv.(hospital)	2.625
Dyspnoea (hospital)	1.693
HR (prehospital)	1.609
Typical chest pain (hospital)	1.439
Atypical chest pain (hospital)	1.439
SBP (prehospital)	1.270
CVRF: Hypertension	0.508
CVRF: Diabetes	0.508
CVRF: known CAD	0.508
Dyspnoea (prehospital)	0.423
Atypical CP (prehospital)	0.339
ThoraxTypical CP (prehospital)	0.339
CKD	0.339
Hb	0.254
Age	0.000
Sex	0.000
CVRF: positive family history	0.000
CVRF: Dyslipidemia	0.000
CVRF: Nicotine abuse	0.000

Model 3	missing values (%)
BMI	40.644
ECG: STE (prehospital)	29.721
ECG: STD (prehospital)	29.721
ECG: T wave inv.(prehospital)	29.721
ECG: LBBB (prehospital)	29.721
Lactate	22.608
Symptom onset	21.423
Typical CP (prehospital)	12.616
Echo: RWMA	8.806
Echo: LVEF category	7.875
ECG: STD (hospital)	2.625
ECG: LBBB (hospital)	2.625
ECG: STE (hospital)	2.625
ECG: T wave inv.(hospital)	2.625
Dyspnoea (hospital)	1.693
HR (prehospital)	1.609
Typical chest pain (hospital)	1.439
Atypical chest pain (hospital)	1.439
LDH	1.270
SBP (prehospital)	1.270
CRP	0.677
CVRF: known CAD	0.508
Creatinine	0.508
CVRF: Diabetes	0.508
CVRF: Hypertension	0.508
Dyspnoea (prehospital)	0.423
CKD	0.339
CK	0.339
Atypical CP (prehospital)	0.339
Typical CP (prehospital)	0.339
Hb	0.254
Leukocytes	0.254
Sex	0.000
CVRF: Nicotine abuse	0.000
CVRF: positive family history	0.000
CVRF: Dyslipidemia	0.000
hsTrop1	0.000
Age	0.000

Model 4	missing values (%)
BMI	40.644
hsTrop2	32.007
ECG: STE (prehospital)	29.721
ECG: STD (prehospital)	29.721
ECG: T wave inv.(prehospital)	29.721
ECG: LBBB (prehospital)	29.721
Lactate	22.608
Symptom onset	21.423
Typical CP (prehospital)	12.616
Echo: RWMA	8.806
Echo: LVEF category	7.875
ECG: STD (hospital)	2.625
ECG: T wave inv.(hospital)	2.625
ECG: LBBB (hospital)	2.625
ECG: STE (hospital)	2.625
Dyspnoea (hospital)	1.693
HR (prehospital)	1.609
Typical chest pain (hospital)	1.439
Atypical chest pain (hospital)	1.439
LDH	1.270
SBP (prehospital)	1.270
CRP	0.677
CVRF: known CAD	0.508
Creatinine	0.508
CVRF: Diabetes	0.508
CVRF: Hypertension	0.508
Dyspnoea (prehospital)	0.423
CKD	0.339
CK	0.339
Atypical CP (prehospital)	0.339
Typical CP (prehospital)	0.339
Hb	0.254
Leukocytes	0.254
Sex	0.000
CVRF: Nicotine abuse	0.000
CVRF: positive family history	0.000
hsTrop1	0.000
CVRF: Dyslipidemia	0.000
Age	0.000

Supplemental Figures

Figure S1

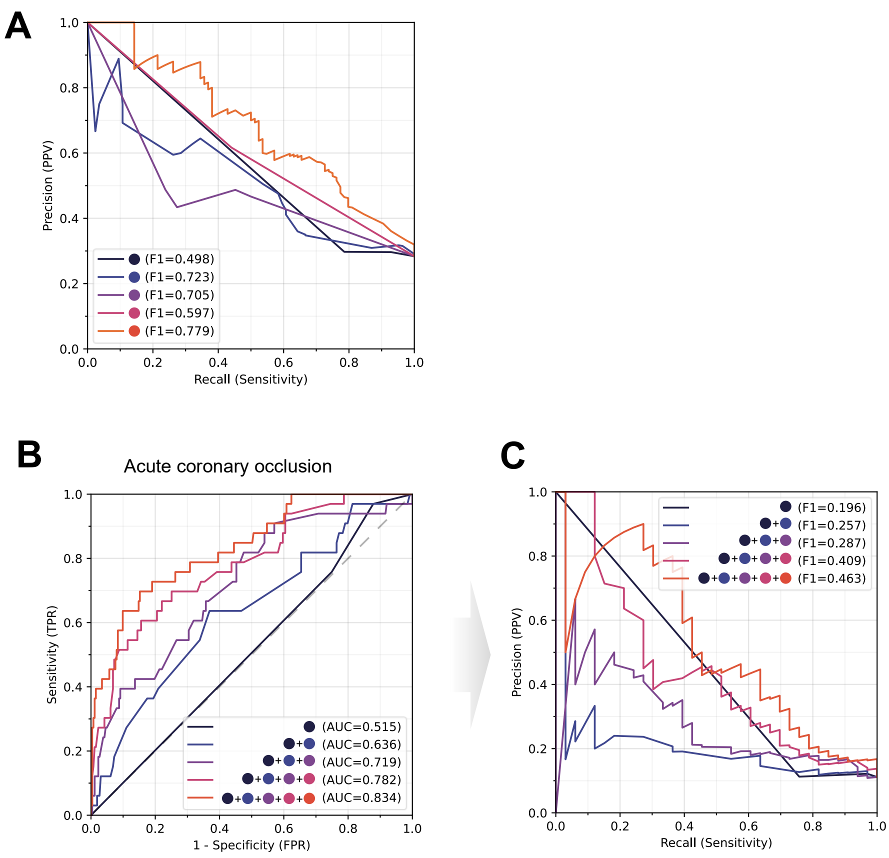

Figure S1: Further analysis of simple logistic regression models

A Precision-Recall curve for logistic regression models with single parameters (corresponding ROC in **Figure 2C**) plots the dependency of recall (sensitivity) and precision (PPV). **B** Prediction of acute coronary occlusion by averaging the predictions from the items of the diagnostic ladder. **C** Corresponding Precision-Recall curve to (**B**). Dotted line represents models including standard troponin testing (Trop) in contrast to high-sensitivity troponin (hsTrop) with a solid line.

ROC – receiver operating characteristics curve, FPR – false positive rate, TRP – true positive rate, PPV – positive predictive value

Figure S2

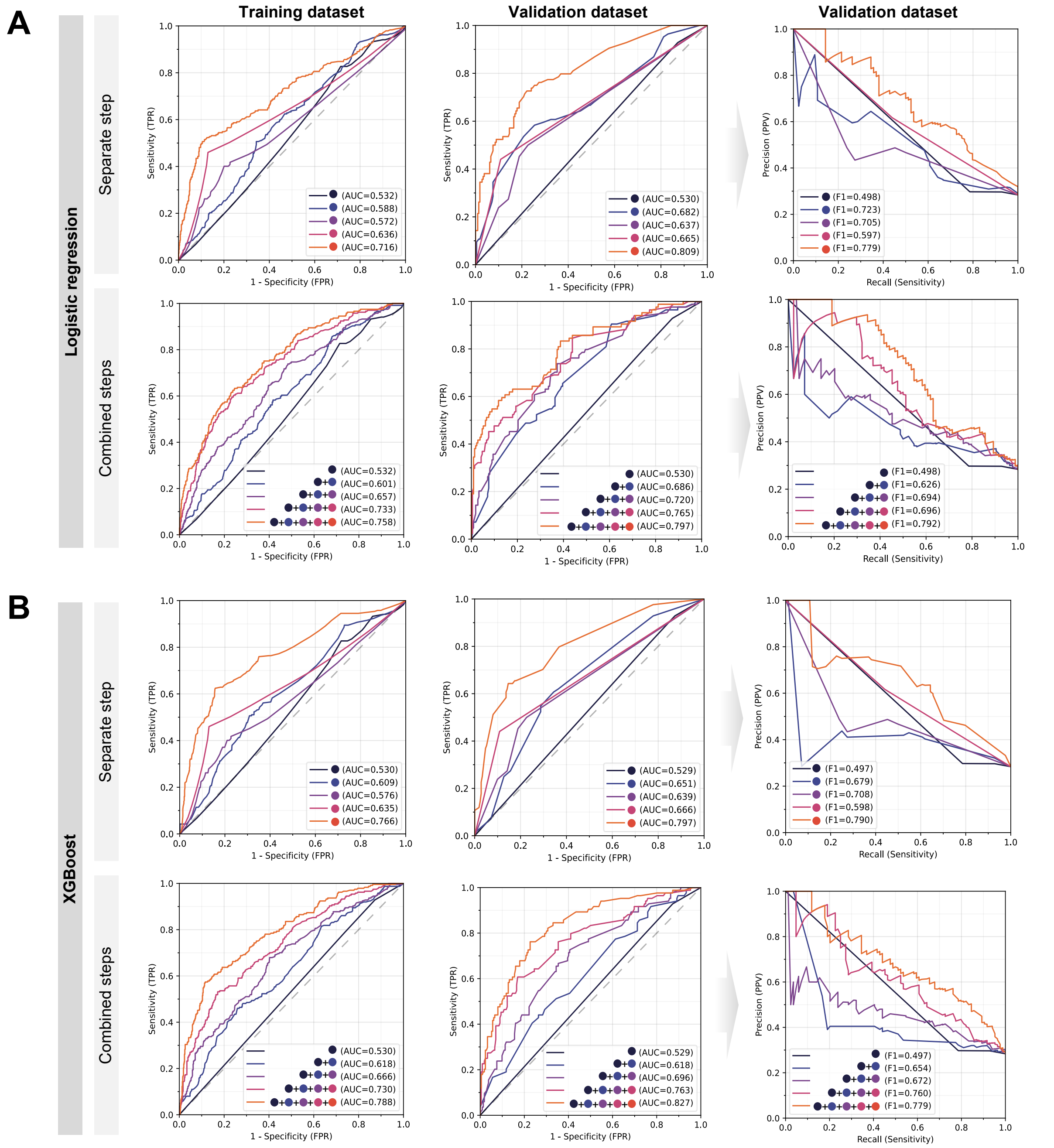

Figure S2: Diagnostic performance of logistic regression vs. XGBoost on the diagnostic ladder items

A ROC curves for the training (left) and validation (center) dataset and the corresponding Precision-Recall-Curve for the validation dataset. Logistic regression was used here.

B ROC curves for the training (left) and validation (center) dataset and the corresponding Precision-Recall-Curve for the validation dataset. XGBoost was used here.

Figure S3

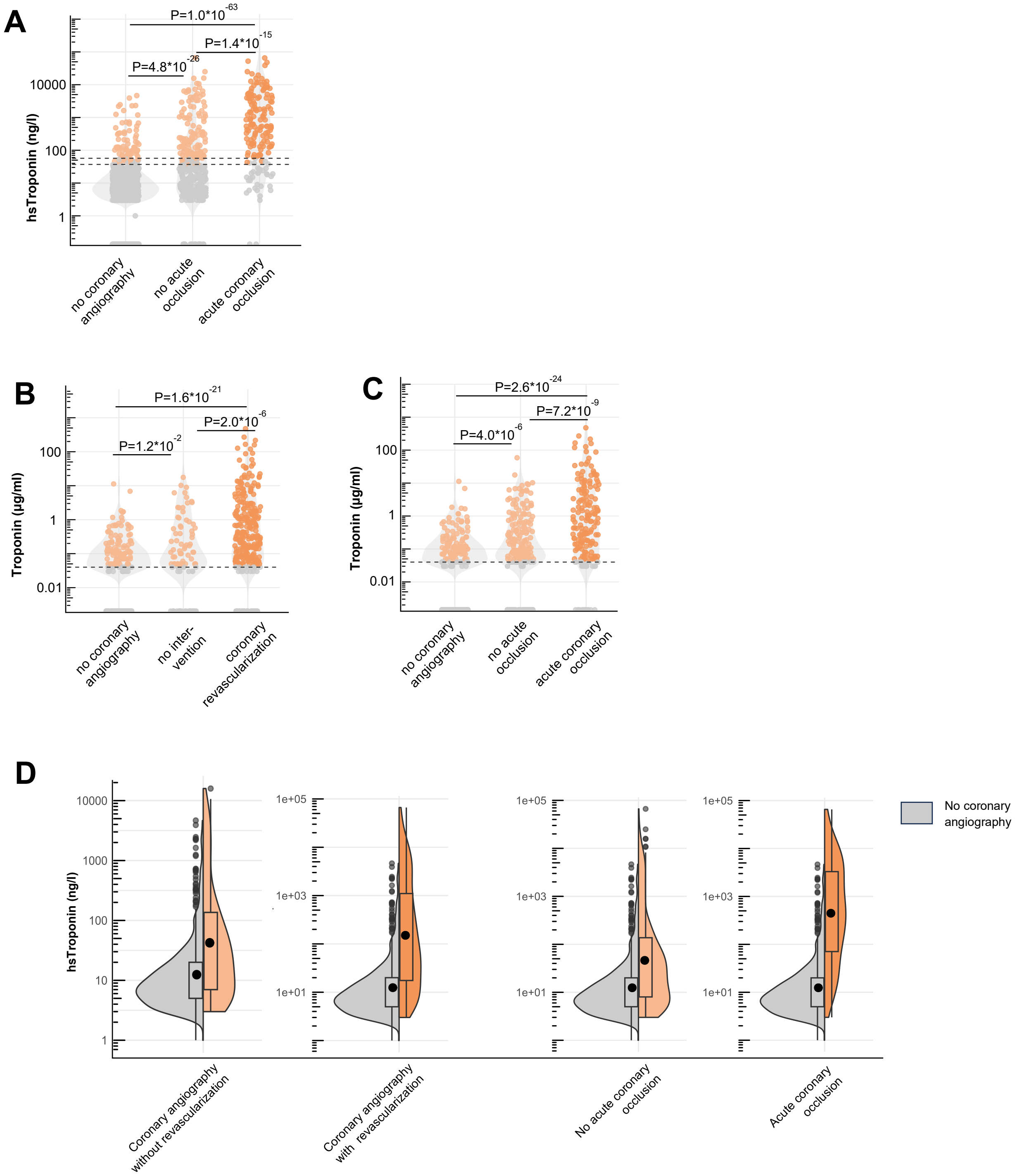

Figure S3: Diagnostic value of standard troponin testing

A Quantification of diagnostic value of high-sensitivity troponin for estimating the need for coronary revascularization or acute coronary occlusion. **B, C** Quantification of diagnostic value of conventional troponin testing for estimating the need for coronary revascularization (**B**) or acute coronary occlusion (**C**). The dotted lines represent the cut-offs for elevated troponin levels, which are sex dependent in case of high-sensitivity troponin. Kruskal-Wallis-test Dunn's test with Holm adjustment for multiple testing. **D** Simplified visualization using split-violin-plots.

Figure S4

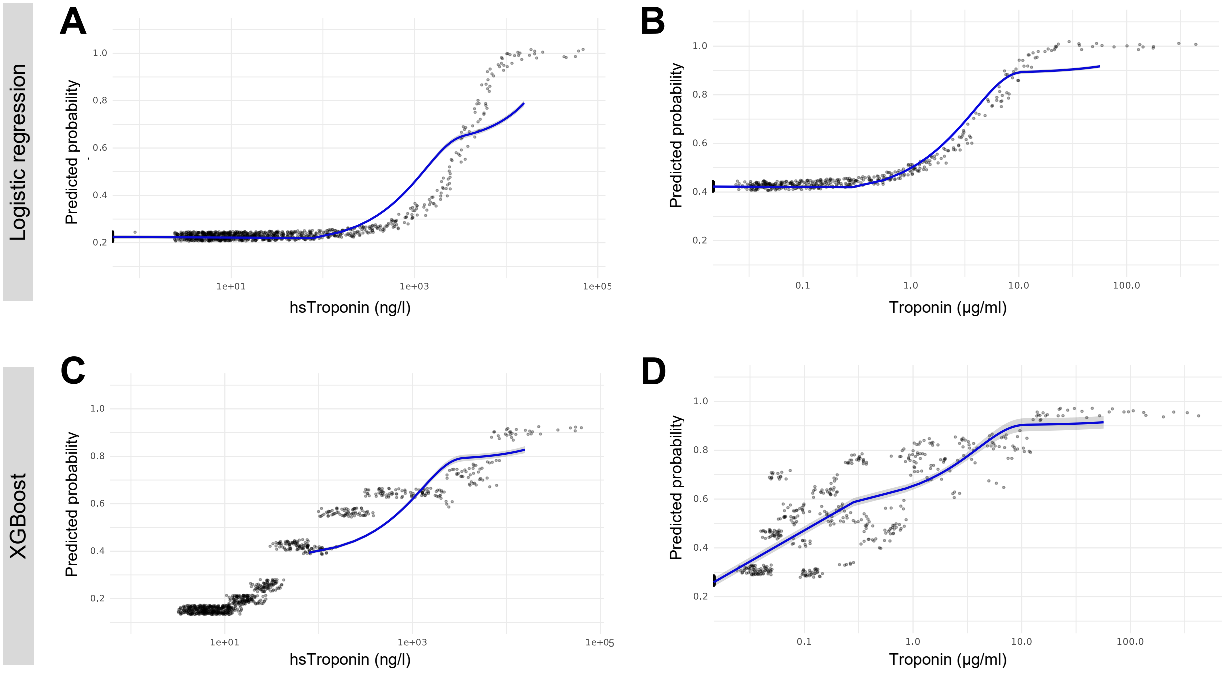

Figure S4: Restricted cubic splines

High-sensitivity (A,C) and standard troponin (B,D) levels were modelled against Logistic Regression- or XGBoost-predicted probability using restricted cubic spline function with three knots placed at the 10th, 50th and 90th percentile.

Figure S5

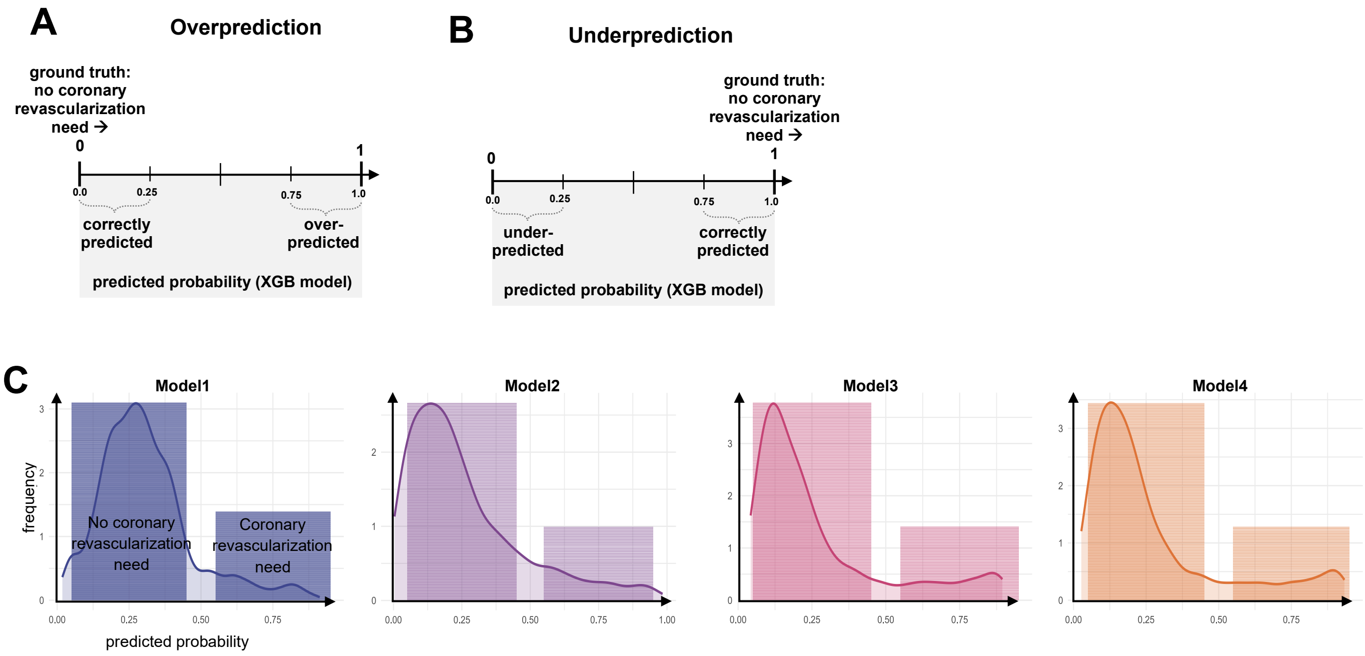

Figure S5: Frequency distributions of predicted probabilities and ground truths per model
A, B Graphical visualization of over- and underprediction definition. **C** Combined histogram and bar plot. The bar plot visualizes the frequency of the ground truth variables 0 (= no coronary revascularization) and 1 (coronary revascularization). The histogram shows the distribution of the predicted probabilities among the different models.

Figure S6

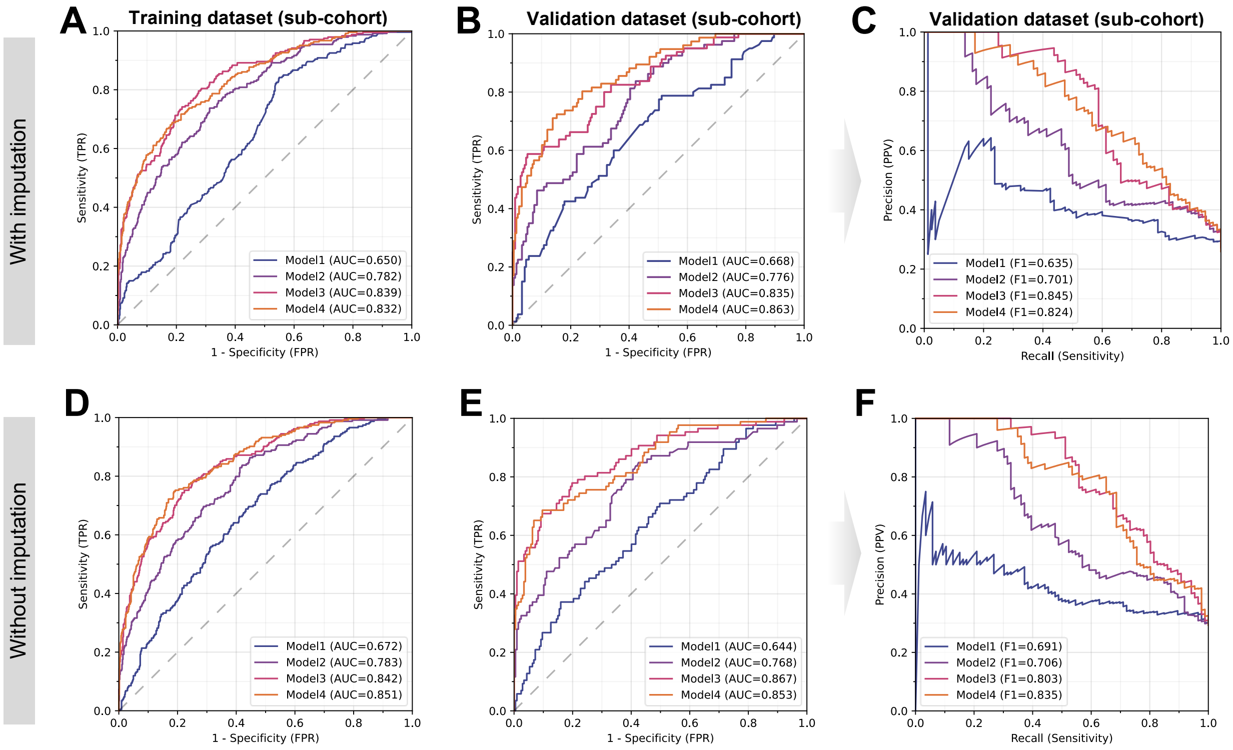

G

		AUC	Accuracy	Precision (weighted)	Recall (weighted)	Specificity	F1 Score (weighted)	AUPRC
With imputation	**Model 1**	0.668 (0.598-0.738)	0.568 (0.513-0.624)	0.721 (0.662-0.779)	0.568 (0.513-0.624)	0.49 (0.425-0.556)	0.635 (0.585-0.685)	0.424 (0.318-0.53)
	**Model 2**	0.776 (0.72-0.832)	0.645 (0.591-0.699)	0.768 (0.72-0.816)	0.645 (0.591-0.699)	0.578 (0.513-0.644)	0.701 (0.655-0.747)	0.61 (0.506-0.714)
	**Model 3**	0.835 (0.784-0.887)	0.846 (0.804-0.888)	0.843 (0.798-0.888)	0.846 (0.804-0.888)	0.949 (0.919-0.978)	0.845 (0.801-0.888)	0.744 (0.663-0.826)
	**Model 4**	0.863 (0.817-0.909)	0.82 (0.775-0.866)	0.827 (0.782-0.872)	0.82 (0.775-0.866)	0.863 (0.818-0.908)	0.824 (0.779-0.868)	0.744 (0.654-0.833)
Without imputation	**Model 1**	0.644 (0.577-0.71)	0.701 (0.649-0.752)	0.681 (0.624-0.739)	0.701 (0.649-0.752)	0.841 (0.792-0.891)	0.691 (0.636-0.745)	0.426 (0.322-0.53)
	**Model 2**	0.768 (0.709-0.828)	0.655 (0.6-0.71)	0.767 (0.719-0.816)	0.655 (0.6-0.71)	0.579 (0.51-0.647)	0.706 (0.659-0.753)	0.645 (0.553-0.737)
	**Model 3**	0.867 (0.82-0.914)	0.793 (0.747-0.839)	0.813 (0.77-0.856)	0.793 (0.747-0.839)	0.804 (0.749-0.858)	0.803 (0.759-0.846)	0.8 (0.732-0.869)
	**Model 4**	0.853 (0.804-0.903)	0.837 (0.793-0.88)	0.834 (0.789-0.879)	0.837 (0.793-0.88)	0.903 (0.862-0.945)	0.835 (0.791-0.88)	0.774 (0.697-0.851)

mean, 95% confidence interval
AUC – area under the curve
AUPRC – area under the precision-recall-curve

Figure S6: Sub-cohort analysis on intersecting patients analyzed in all four XGBoost models

A subcohort with identical patients from all XGBoost models was derived and analyzed for its performance. Receiver operating characteristics for the training (**A**) and validation (**B**) subcohort. **C** Precision-recall curve for validation dataset from subcohort. **D** Table with performance metrics, to be compared with Supplemental Table 1.

Figure S7

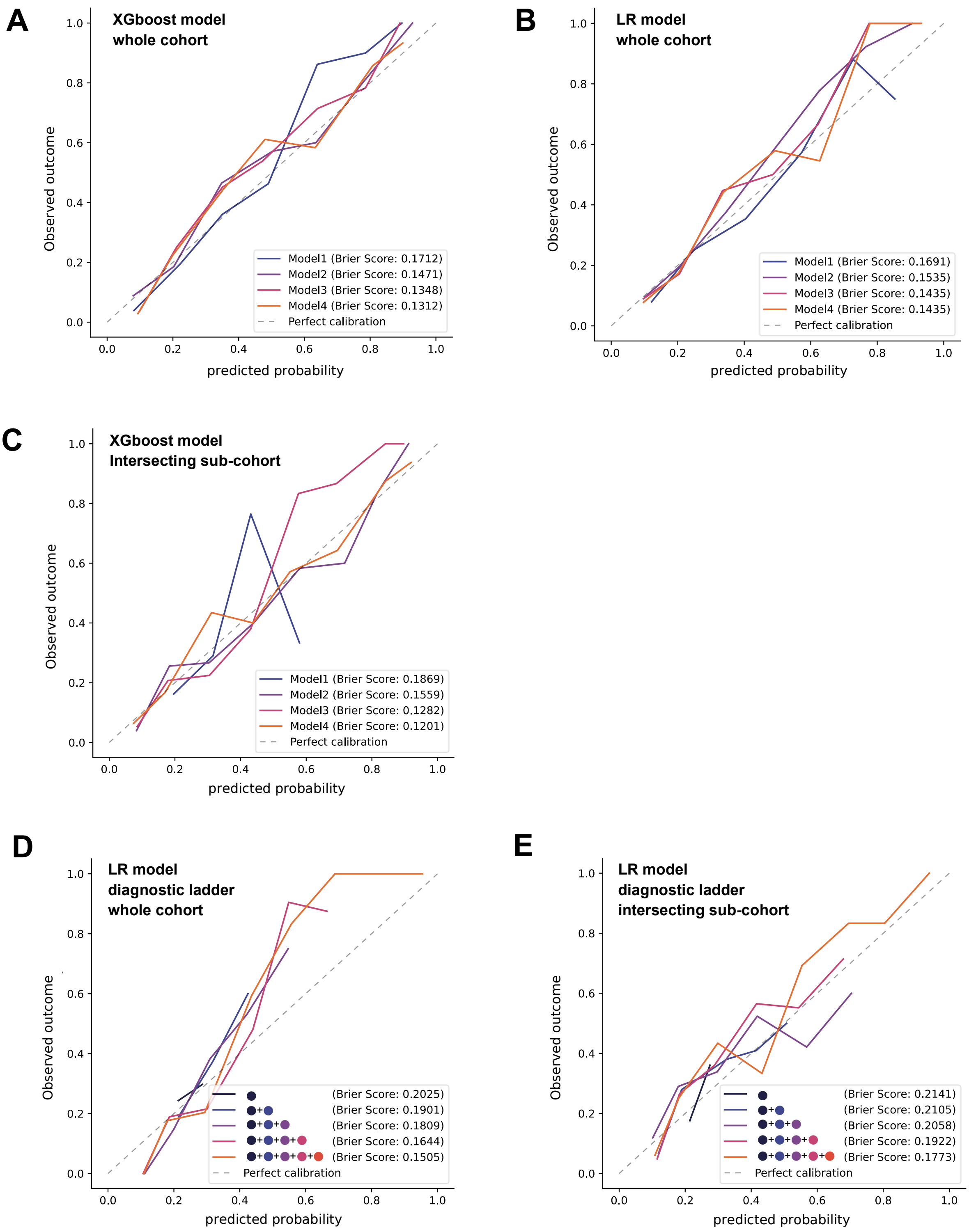
A
XGboost model
whole cohort
Observed outcome
predicted probability
Model1 (Brier Score: 0.1712)
Model2 (Brier Score: 0.1471)
Model3 (Brier Score: 0.1348)
Model4 (Brier Score: 0.1312)
Perfect calibration
B
LR model
whole cohort
Observed outcome
predicted probability
Model1 (Brier Score: 0.1691)
Model2 (Brier Score: 0.1535)
Model3 (Brier Score: 0.1435)
Model4 (Brier Score: 0.1435)
Perfect calibration
C
XGboost model
Intersecting sub-cohort
Observed outcome
predicted probability
Model1 (Brier Score: 0.1869)
Model2 (Brier Score: 0.1559)
Model3 (Brier Score: 0.1282)
Model4 (Brier Score: 0.1201)
Perfect calibration
D
LR model
diagnostic ladder
whole cohort
Observed outcome
predicted probability
(Brier Score: 0.2025)
(Brier Score: 0.1901)
(Brier Score: 0.1809)
(Brier Score: 0.1644)
(Brier Score: 0.1505)
Perfect calibration
E
LR model
diagnostic ladder
intersecting sub-cohort
Observed outcome
predicted probability
(Brier Score: 0.2141)
(Brier Score: 0.2105)
(Brier Score: 0.2058)
(Brier Score: 0.1922)
(Brier Score: 0.1773)
Perfect calibration

F

		Brier-Score	MSE from perfect calibration	Intercept	Slope
XGboost model whole cohort	Model 1	0.1712	0.0097	-0.0711	1.2343
	Model 2	0.1471	0.0082	0.0438	0.9796
	Model 3	0.1348	0.0119	0.0177	1.0683
	Model 4	0.1312	0.0079	0.0108	1.0525
LR model whole cohort	Model 1	0.1691	0.0301	0.0091	0.9393
	Model 2	0.1535	0.0094	-0.0237	1.1967
	Model 3	0.1435	0.0102	-0.0240	1.1720
	Model 4	0.1435	0.0114	-0.0212	1.1439
XGboost model Intersecting sub-cohort	Model 1	0.1869	0.0434	0.1211	0.6998
	Model 2	0.1559	0.0040	-0.0244	1.0343
	Model 3	0.1282	0.0179	-0.0653	1.2698
	Model 4	0.1201	0.0027	0.0157	0.9915
LR model diagnostic ladder whole cohort	Model 1	0.2025	0.0005	0.0850	0.7414
	Model 2	0.1901	0.0115	-0.2340	1.9491
	Model 3	0.1809	0.0148	-0.1850	1.7215
	Model 4	0.1644	0.0319	-0.1896	1.6923
	Model 5	0.1505	0.0317	-0.0609	1.3115
LR model diagnostic ladder intersecting sub-cohort	Model 1	0.2141	0.0044	-0.4715	3.0240
	Model 2	0.2105	0.0028	0.0225	0.9789
	Model 3	0.2058	0.0096	0.1282	0.6715
	Model 4	0.1922	0.0066	0.0236	1.0624
	Model 5	0.1773	0.0098	0.0176	1.0711

Figure S7: Calibration plots

Calibration plots with uniform binning showing Model1 to Model 4 using XGBoost (**A**) or Logistic Regression (**B**) as model, subsetted on the intersecting sub-cohort (**C**). Fig. (**D**) and (**E**) show calibration for the logistic regression models on the diagnostic ladder items, on the full cohort (the same as for Model4) and the intersecting sub-cohort. **F** Detailed calibration measures, including Brier score, mean square error (MSE) from perfect calibration, and intercept/slope for linear regression.

Figure S8

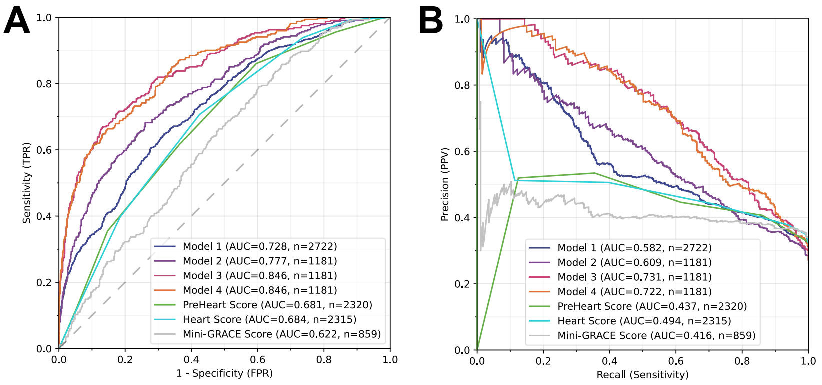

C

		Model 1	Model 2	Model 3	Model 4
Binary NRI	PreHeart Score	0.0332	0.0365	0.0698	0.0696
	Heart Score	0.0028	0.0369	0.0702	0.07
	Mini-GRACE Score	0.0346	0.0615	0.0941	0.0939
Continuous NRI	PreHeart Score	0.0181	0.119	0.1986	0.2278
	Heart Score	-0.0247	0.0816	0.1464	0.1725
	Mini-GRACE Score	0.1383	0.1733	0.2416	0.2518

Figure S8: Comparison with other scores

Comparison of XGBoost models 1-4 with PreHeart score, Heart score and mini-GRACE score, including specification of sample sizes due to missing values. Representation as receiver-operating characteristics (**A**) and precision-recall curve (**B**). **C** Quantification the ML model's benefit using binary and continuous Net reclassification index (NRI).

Figure S9

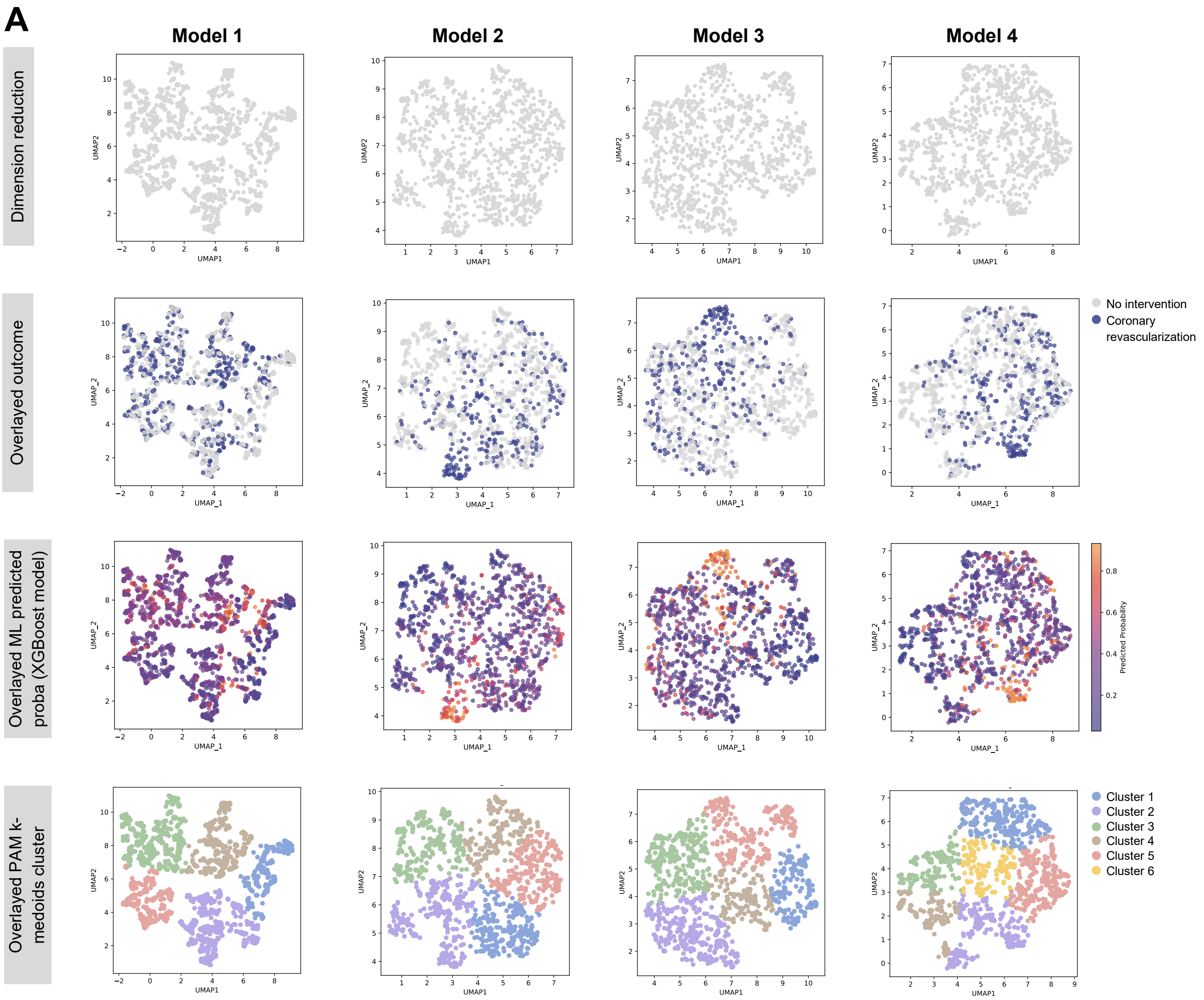

Figure S9: Phenotypization of patient cohort using unsupervised machine learning

A Overview panel for patient cohorts analyzed in XGBoost models 1-4, using the described variables each. First row displays dimension reduction on variables described per model, applying Gower as distance metric and using UMAP for dimension reduction. Second row highlights patients with need for coronary revascularization. Third row shows the predicted probability for coronary revascularization need, as calculated using the XGBoost models. The last rows shows partitioning-around-medoids (PAM)-identified clusters.

Figure S10

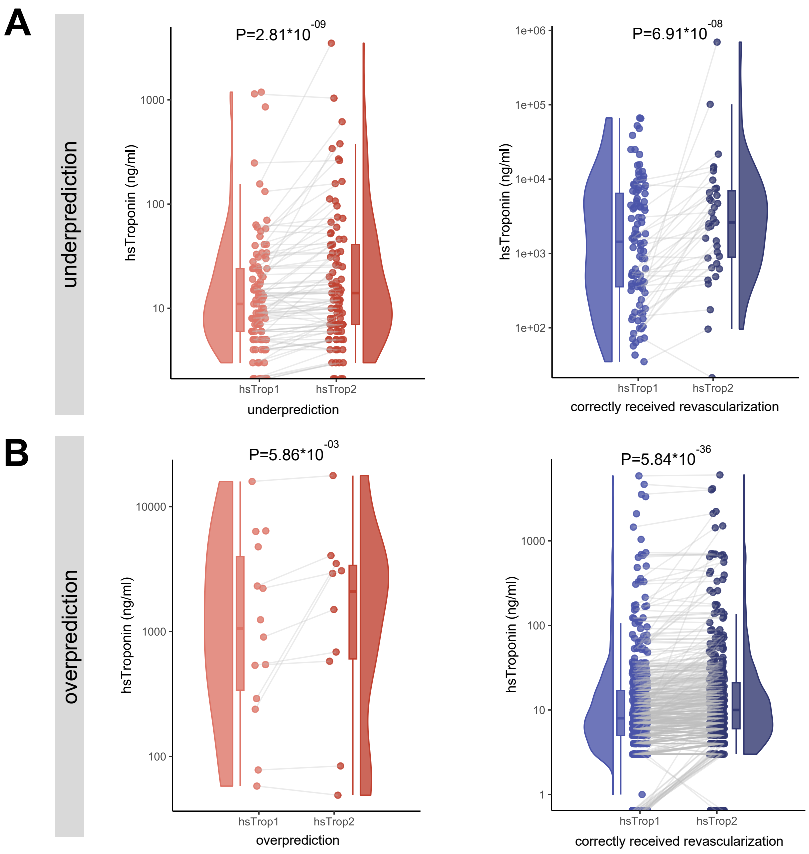

Figure S10: Sequential troponin testing in misclassified patients.

Before-after plots comparing the initial high-sensitivity Troponin (hsTrop1) with the sequential high-sensitivity Troponin (hsTrop2) in underpredicted (**A**) or overpredicted (**B**) patients in red, in comparison to the high-sensitivity Troponin dynamics in correctly classified patients (in dark blue). Paired Wilcox test.
